# Scanxiety and Fear of Recurrence in Young Adult Female Breast and Gynaecological Cancer Survivors: Investigating Shared Mechanisms

**DOI:** 10.1002/pon.70050

**Published:** 2024-12-18

**Authors:** Diya S. Patel, Sarah N. Webster, Emily J. Dowling, Claudia R. Knowles, Georgina Lockwood‐Taylor, Daelin Coutts‐Bain, Laura E. Simons, Elisabeth J. Diver, Joseph Chilcot, Lidia Schapira, Lauren C. Heathcote

**Affiliations:** ^1^ Health Psychology Section Department of Psychology Institute of Psychiatry Psychology, and Neuroscience King's College London London UK; ^2^ Department of Psychology University of Miami Miami Florida USA; ^3^ The School of Psychology University of Sydney Sydney Australia; ^4^ Department of Anesthesiology Perioperative, and Pain Medicine Stanford University School of Medicine Stanford California USA; ^5^ Department of Obstetrics and Gynecology Division of Gynecologic Oncology Stanford University School of Medicine Stanford California USA; ^6^ Department of Medicine Division of Medical Oncology Stanford University Stanford California USA

**Keywords:** adolescent and young adults (AYAs), bodily threat monitoring, breast cancer, cancer, cancer survivors, fear of cancer recurrence, gynaecological cancer, oncology, quality of life, scanxiety

## Abstract

**Background:**

Adolescent and young adult (AYA) females are vulnerable to psychological sequelae following cancer diagnosis and treatment. Fear of cancer recurrence (FCR) is well‐documented in cancer survivors, however AYA survivors of breast and gynaecological cancers are less well‐studied. Moreover, little is known about scan‐related fears and anxiety (‘scanxiety’) in survivors of any age group.

**Aims:**

This study aimed to assess demographic, medical, and quality‐of‐life correlates of FCR and scanxiety in AYA female breast and gynaecological cancer survivors post‐treatment. Additionally, we explored potential shared mechanisms of FCR and scanxiety, including intolerance of uncertainty, bodily threat monitoring, and perceived stress.

**Methods:**

AYA breast and gynaecological cancer survivors (*N* = 115) completed measures of FCR, scanxiety, intolerance of uncertainty, bodily threat monitoring, perceived stress, and quality of life. Bivariate associations and a structural equation model explored relationships between these variables.

**Results:**

Both FCR and scanxiety were prevalent, with 84% reporting clinically meaningful FCR and 38% reporting severe FCR. Higher FCR and scanxiety were both associated with poorer quality of life. FCR and scanxiety were moderately associated but not entirely overlapping. Intolerance of uncertainty, bodily threat monitoring, and perceived stress were significantly correlated with both FCR and scanxiety. The structural equation model indicated that bodily threat monitoring is a plausible intermediate variable linking intolerance of uncertainty and FCR, but not scanxiety.

**Conclusions:**

FCR and scanxiety are common in AYA survivors of breast and gynaecological cancers, with potentially distinct underlying mechanisms. Interventions targeting intolerance of uncertainty and bodily threat monitoring may reduce FCR, while further research is needed to identify therapeutic targets for scanxiety.

## 1 Introduction

Adolescents and young adults (AYAs, 18–39 years) are at heightened risk of experiencing mental health sequalae following a cancer diagnosis and treatment, particularly anxiety and fear of cancer recurrence (FCR) [1, 2]. Females also show heightened FCR compared to males [3]. Breast and gynaecological cancers account for 25% of the cancers most commonly seen in AYA females [4], yet there have been few studies of FCR in AYA survivors of breast cancer [1, 5, 6, 7], and very few in AYA survivors of gynaecological cancers, despite both representing at‐risk groups.

In addition to generalised fears, worries, and concerns about the possibility of cancer returning (FCR), there is growing recognition that cancer survivors experience significant fear and anxiety around routine surveillance tests and scans [8]. ‘Scanxiety’ has been defined as distress and/or anxiety occurring before, during, and after cancer‐related imaging and scans [8]. Scanxiety can arise in part as an acute manifestation of FCR as scans hold implications for the possibility of cancer having returned; however, other components of scanxiety have been described, including anxiety related to scan procedures (e.g., claustrophobia, physical discomfort) [8]. A recent systematic review [8] found that scanxiety tended to be higher among females, those with less time since diagnosis, lower education levels, and greater baseline general anxiety. Elevated scanxiety was also found to be associated with greater somatic symptom burden and poorer quality of life [8]. However, few of the included studies in this review were in AYA survivors and thus little is known about who is at risk for scanxiety or its quality‐of‐life consequences among younger survivors.

Identifying risk and maintaining factors of FCR and scanxiety across cancer survivor populations, including in AYAs, will be important to develop clinical intervention strategies. To date, there are no published comprehensive theoretical models for scanxiety. However, given their conceptual overlap, theoretical models of FCR offer putative mechanisms that may also confer risk for the development and persistence of scanxiety [9, 10, 11]. Uncertainty is inherent in cancer survivorship, and the extent to which an individual can tolerate uncertainty may have implications for both FCR and for scanxiety. After finishing cancer treatment, there are uncertainties around the possibility of relapse, the meaning of physical symptoms, and the consequences of relapse for further treatment and survival. A robust body of research has shown that intolerance of uncertainty is a cognitive vulnerability factor for worry and a maintaining factor for Generalized Anxiety Disorder (GAD) [12, 13, 14]. There is emerging research that intolerance of uncertainty also acts as a dispositional risk factor for anxiety in health contexts [15], including cancer [9]. Moreover, the Cancer Threat Interpretation (CTI) Model [11] proposes that pain and other bodily symptoms are salient triggers for uncertainty and fear in the context of cancer survival. Individuals, particularly those high in intolerance of uncertainty, may excessively monitor their bodily symptoms—termed ‘bodily threat monitoring’ [16]—as an uncertainty‐reducing behaviour. As an unintended consequence, bodily threat monitoring may increase worry about benign bodily symptoms, thereby maintaining FCR over time [11, 17]. Those with greater scanxiety may also engage in greater bodily threat monitoring around the time of a scan to pre‐empt possible bad news, a form of bracing behaviour [17]. Thus, bodily threat monitoring may act as a mechanism linking intolerance of uncertainty with both elevated FCR and scanxiety.

Stress is another putative mechanism that may link intolerance of uncertainty with elevated FCR and scanxiety. That is, intolerance of uncertainty may heighten general stress levels within the inherently uncertain context of cancer survivorship, and in turn, perceived stress may exacerbate concerns about being able to cope with and manage a possible cancer recurrence. While the uncertainties inherent in cancer survival may wax and wane in everyday life, they can come to the fore during the stressful waiting period before a routine cancer surveillance scan [18]. Drawing on stress and coping theories that emphasize subjective stress appraisal (e.g., [19]), perceived stress is a well‐documented contributor to anxiety and emotional distress, making it a plausible mechanism linking the experience of cancer‐related threats to both FCR and scanxiety. Altogether, FCR theory and empirical evidence indicates that intolerance of uncertainty may act as a dispositional risk factor for both FCR and scanxiety, with bodily threat monitoring and perceived stress as putative maintaining mechanisms.

This study had two aims. First, we aimed to assess the demographic, medical, and quality‐of‐life correlates of FCR and scanxiety in AYA female breast and gynaecological cancer survivors aged 18–39 years who have completed active cancer treatment. We focus on experiences of scanxiety in the run up to cancer surveillance scans and medical tests. We hypothesised that AYA survivors who reported greater FCR and scanxiety would report elevated reassurance‐seeking behaviours and worse quality of life. We did not have directional hypotheses regarding demographic or medical correlates of scanxiety and FCR. Drawing from FCR theory, we additionally aimed to examine potential shared mechanisms of FCR and scanxiety in this population. As a starting point for empirical investigation, we examined bivariate associations between FCR, scanxiety, intolerance of uncertainty, bodily threat monitoring, and perceived stress. We hypothesised that AYA survivors who reported greater FCR would also report greater scanxiety, and that higher levels of both FCR and scanxiety would be associated with greater intolerance of uncertainty, bodily threat monitoring, and perceived stress. We also hypothesised that within a structural equation model, intolerance of uncertainty would directly associate with FCR and scanxiety and be indirectly associated with these outcomes via bodily threat monitoring and perceived stress.

## 2 Methods

### 2.1 Participants and Procedure

The data reported in this manuscript are from a longitudinal survey of AYA (18–39 years) survivors of breast and gynaecological cancer; baseline data related to the study aims are reported here. For inclusion, AYAs (aged 18–39 years) who had completed treatment with curative intent for breast cancer (*N* = 81) or gynaecological cancer (*N* = 34; including ovarian, cervical, and endometrial cancers) completed baseline surveys. Exclusion criteria were evidence of metastatic disease (stage IV), significant cognitive impairment as self‐reported or reported by the clinician, and non‐English speaking. AYAs receiving adjuvant hormonal therapies were not excluded. Participants were purposefully recruited through clinical and community networks to increase sample diversity; this included the Stanford Women's Cancer Centre, the Bay Area Young Adult Survivors cancer organisation, and Facebook advertising (Facebook‐recruited participants were required to live in the US). Participants provided written informed consent for participating in the study and were compensated for their time completing the survey with a $10 Amazon Voucher and additionally entered a raffle for a $250 Amazon Voucher. Ethical approval was obtained by the Stanford Medicine Institutional Review Board (IRB‐44463).

### 2.2 Measures

Participants responded to demographic questions and reported their medical history including cancer type, cancer stage, time since first diagnosis and treatment completion, scan frequency, and recurrence history.

### 2.3 Scanxiety

Scanxiety was assessed using an adapted version of the Impact of Events Scale—Revised (IES‐R) [20]. The IES‐R is one of the most widely used instruments to measure the psychological impact of a specific event and adapted versions have previously been used to assess scanxiety in cancer survivor populations [21, 22]. For this study, we used the wording and number of items from the youth‐adapted measure of the IES‐R (CRIES‐8) [23]; items in the CRIES‐8 are almost identical to items in the IES‐R but with slightly simpler wording which was deemed preferable by clinician and patient partners. The CRIES‐8 items have been used previously in young adult populations including those aged over 18 years [17] and were thus considered appropriate for use in this study. Instructions read ‘Below is a list of difficulties people sometimes have around stressful life events. Please indicate how frequently each statement was true for you in the run up to your most recent scan or cancer check‐up’. Scoring adhered to the CRIES‐8 validation paper [23] in that items were scored as 0 (not at all), 1 (rarely), 3 (sometimes), and five (often). In this study, total scores could range from 0–40 with higher scores indicating greater scanxiety (*α* = 0.79 in this sample).

### 2.4 Fear of Cancer Recurrence

FCR severity was measured with the Fear of Cancer Recurrence Inventory ‐ Severity Subscale (FCRI‐SF), assessing the presence and severity of FCR‐related intrusive thoughts [24]. The FCRI‐SF comprises nine‐items, rated on a five‐point Likert scale ranging from 0 (not at all like me) to 4 (a great deal like me). Established clinical cut‐offs for the FCRI‐SF denote that scores ≥ 13 indicate clinically meaningful FCR and scores ≥ 22 indicate clinically severe FCR [25].

Total scores can range from 0–36, with higher scores indicating greater FCR (*α* = 0.83 in this sample).

### 2.5 Reassurance‐Seeking Behaviours

The FCRI‐reassurance subscale includes three‐items evaluating participants' use of coping strategies as means to seek reassurance for their FCR [24]. Items are rated on a five‐point Likert scale ranging from 0 (not at all like me) to 4 (a great deal like me). Total scores can range from 0–12, with higher scores indicate greater reassurance seeking (*α* = 0.75 in this sample).

### 2.6 Intolerance of Uncertainty

The 12‐item Intolerance of Uncertainty Scale (IUS‐12) assesses one's ability to tolerate uncertain and ambiguous situations [26].Participants respond on a five‐point scale ranging from 1 (not at all characteristics of me) to five (entirely characteristic of me). Total scores can range from 12–60, with higher scores indicate greater intolerance of uncertainty (*α* = 0.92 in this sample).

### 2.7 Bodily Threat Monitoring

The 19‐item Bodily Threat Monitoring Scale (BTMS) was used to assess tendencies to monitor and interpret bodily sensations as threatening [16]. Items are rated on a five‐point Likert scale ranging from 0 (not at all like me) to four (entirely like me). Total scores can range from 0–76 and higher scores indicate greater bodily threat monitoring (*α* = 0.95 in this sample).

### 2.8 Perceived Stress

The Perceived Stress Scale (PSS) comprises 10 items that capture the degree to which participants have perceived life as unpredictable, overloading, and uncontrollable in the past month [27]. Items are rated on a five‐point scale ranging from 0 (never) to 4 (very often). Total scores could range from 0–40, with higher scores indicating greater perceived stress (*α* = 0.85 in this sample).

### 2.9 Health‐Related Quality of Life

The 16‐item McGill Quality of Life Questionnaire (MQoL) evaluates Health‐Related Quality of Life (HRQoL) across 4 domains: physical symptoms, psychological symptoms, existential well‐being, and social support [28]. Participants rated how true each item was to them on a 10‐point scale ranging from 0–10. Higher mean MQoL and subscale scores indicate better HRQoL. The MQoL total score and its subscales demonstrated good internal consistency in this sample (total score, *α* = 0.90; physical symptoms, *α* = 0.92; psychological symptoms, *α* = 0.89; existential well‐being, *α* = 0.88; social support, *α* = 0.81).

### 2.10 Statistical Methods

Data were inspected for normality and completeness. Mean substitution was used when missing data for individual participants was ≤ 10%; where missing data was > 10% the case was disregarded for that variable. Descriptive statistics and bivariate associations among variables were inspected using SPSS version 28 [29]. The distribution of scanxiety and FCR were summarised with boxplots and scatterplots using R 4.3.2 [30]. Structural equation modelling (SEM) was used to examine shared mechanisms of scanxiety and FCR using AMOS version 28. Including both FCR and scanxiety in the same SEM model was intended to capture their shared mechanisms and interrelated pathways within a unified framework. This approach enables investigation of whether certain mechanisms could contribute to both outcomes simultaneously or whether they act through distinct or overlapping pathways, providing a more comprehensive understanding of their relationship. Individual paths as well as overall model fit indices were inspected, with Chi‐square (χ2) *p* > 0.05, Comparative Fit Index (CFI) > 0.95, Root Mean Square Error of Approximation (RMSEA) < 0.08 with examination of the RMSEA 90%CI, and Tucker‐Lewis Index (TLI) > 0.95 used as cut‐offs to indicate good overall model fit [31, 32]. Of note, although the sample size in this study is below the *N* = 200 threshold where RMSEA can become less reliable, we opted to include it alongside other fit indices (CFI, TLI) to provide a more balanced evaluation of model fit.

## 3 Results

In total, 115 AYA survivors of breast and gynaecological cancers were recruited into the study and completed an online REDCap [33] survey between 2021 and 2022. One participant was excluded from analyses due to being aged > 39 years at survey completion, and three participants were excluded from analyses as they reported evidence of metastatic disease (stage IV), thus yielding 111 participants included in analyses (see Supporting Information S1: Figure S1 for CONSORT diagram). As can be seen in Table 1, participants ranged from 21 to 39 years old (*M* = 34 years) at the time of the study. Two‐thirds of the sample had a history of breast cancer (67.6%), while one‐third had a history of gynaecological cancer (32.4%). Most participants reported having Stage I or II disease at their first diagnosis, and participants were on average 4 years since their diagnosis and 2 years since completing treatment.

**TABLE 1:** Demographics and medical characteristics of the sample.

	Full sample *N* (%)	*M*(SD)	Mdn (range)
Age		34.2 (3.9)	35.0 (21–39)
21–25	3 (2.7)		
26–30	16 (14.4)		
31–39	89 (80.1)		
Ethnicity			
Hispanic, Latino/a, or Spanish origin	17 (15.3)		
Not Hispanic, Latino/a, or Spanish origin	94 (84.7)		
Race			
African American/Black	7 (6.3)		
American Indian/Alaska Native	2 (1.8)		
Asian/Asian American	21 (18.9)		
Caucasian/White	61 (55.0)		
Hawaiian Native or Pacific Islander	3 (2.7)		
Mixed race	8 (7.2)		
Other	6 (5.4)		
Prefer not to say	3 (2.7)		
Highest level of education			
Did not complete high school	1 (0.9)		
Completing/completed high school	12 (10.8)		
Completing/completed an associates degree	11 (9.9)		
Completing/completed a Bachelor's degree	47 (42.3)		
Completing/completed a Master's degree	29 (26.1)		
Completing/completed an education beyond a Master's degree	8 (7.2)		
Other	3 (2.7)		
Employment status			
Student	2 (1.8)		
Unemployed	19 (17.1)		
Part‐time employed	12 (10.8)		
Full‐time employed	76 (68.5)		
Household annual income			
Less than $30,000	12 (10.8)		
$30,000–$59,999	18 (16.2)		
$60,000–$89,999	14 (12.6)		
$90,000–$119,999	22 (19.8)		
$120,000–$149,999	16 (14.4)		
$150,000–$179,999	6 (5.4)		
$180,000–$209,999	4 (3.6)		
Greater than $210,000	17 (15.3)		
Cancer diagnosis			
Breast	75 (67.6)		
Gynaecological	36 (32.4)		
Cancer stage at first diagnosis			
0 (DCIS)	3 (2.7)		
I	42 (37.8)		
II	46 (41.4)		
III	18 (16.2)		
No stage/unknown	2 (1.8)		
Years since diagnosis		4.2 (3.3)	2.8 (0.4–13.5)
Years since treatment completion		2.2 (2.4)	1.6 (0.01–13.2)
History of recurrence			
Yes	8 (7.2)		
No	100 (90.1)		
Adjuvant therapy			
Yes	43 (39%)		
No	68 (61%)		
Frequency of surveillance tests/scans			
Every 1–3 months	31 (27.9)		
Every 4–6 months	47 (42.3)		
Every 7–11 months	2 (1.8)		
Every year	12 (10.8)		
Less than once per year	10 (9.0)		
No longer have tests/scans	9 (8.1)		
Last surveillance test/scan			
0–3 months ago	69 (62.2)		
4–6 months ago	24 (21.6)		
7–12 months ago	8 (7.2)		
13–24 months ago	6 (5.4)		
More than 24 months ago	4 (3.6)		

*Note:* Percentages (1 decimal place) may not sum up to 100% (*N* = 111) due to missing data.

### 3.1 FCR and Scanxiety

**FIGURE 1:**
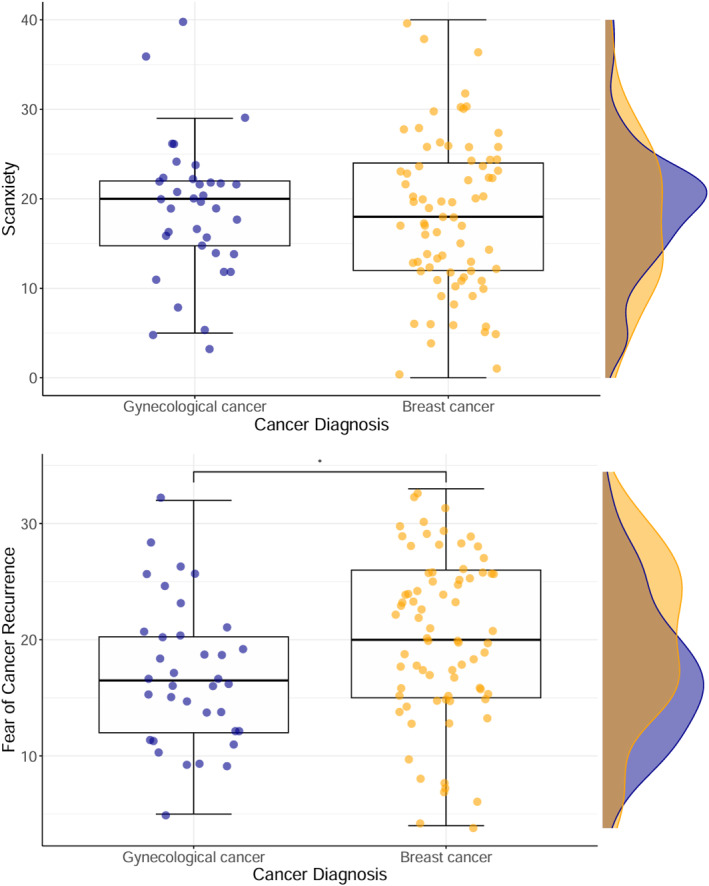
Boxplots and marginal density plots illustrating the range of scanxiety and FCR symptoms across cancer types. *p < 0.05.

As seen in Figures 1A and 1B, there was a wide range of scanxiety and FCR levels reported by both breast and gynaecological cancer survivors. All but one participant (99%) reported some degree of scanxiety as indicated by a score greater than zero. Two participants (2%) yielded the maximum scanxiety score of 40, indicating very high levels of scanxiety. Of note, there are no established cut‐offs for clinical levels of scanxiety. Based on two established clinical cut‐offs for the FCRI‐SF, 84% (95% Confidence Interval: 78%–91%) of the sample scored above the threshold for clinically meaningful FCR (scores ≥ 13) and 38% (95% Confidence Interval: 30%–47%) scored above the threshold for clinically severe FCR (scores ≥ 22).

### 3.2 Demographic and Medical Correlates of FCR, Scanxiety, and HRQoL

As seen in Table 3, breast cancer survivors reported greater FCR and lower overall HRQoL compared to gynaecological cancer survivors. There was no significant difference in scanxiety levels between cancer types (breast vs. gynaecological). Participants with a history of breast cancer were slightly older than those with a history of gynaecological cancer (*M*difference = 2.1 years). All findings hereafter are presented for both diagnostic groups combined. As seen in Table 3, participants who were diagnosed more recently, and those who finished treatment more recently, reported significantly greater FCR severity; scanxiety was not associated with time since diagnosis or treatment completion.

**TABLE 2:** Descriptive statistics and Pearson correlations across psychological variables.

		*M*(SD)	1	2	3	4	5	6	7	8	9	10
Psychological variables	1 Scanxiety	18.28 (8.36)										
	2 FCR severity	19.19 (6.87)	0.434***									
	3 FCR reassurance seeking	3.93 (3.14)	0.247*	0.103								
	4 Bodily threat monitoring	34.47 (16.81)	0.244*	0.543***	0.283**							
	5 Intolerance of uncertainty	31.08 (10.63)	0.278**	0.357***	0.266**	0.496***						
	6 Perceived stress	17.68 (6.50)	0.253**	0.373***	0.066	0.440***	0.633***					
Health‐related quality of life	7 HRQoL total	7.02 (1.59)	−0.244*	−0.343***	0.007	−0.224*	−0.232*	−0.456***				
	8 Physical symptoms	6.04 (3.28)	−0.087	−0.247**	0.104	−0.165	−0.093	−0.248**	0.716***			
	9 Psychological symptoms	6.85 (2.68)	−0.187	−0.438***	0.011	−0.363***	−0.365***	−0.520***	0.738***	0.488***		
	10 Existential wellbeing	7.58 (1.59)	−0.221*	−0.091	−0.078	−0.114	−0.233*	−0.383***	0.633***	0.147	0.347***	
	11 Social support	7.80 (1.96)	−0.195*	−0.001	0.004	0.070	0.156	−0.205*	0.503***	0.044	0.200*	0.625***

*Note:* Correlations reported to 3 decimal places.

Abbreviations: FCR, Fear of Cancer Recurrence; HRQoL, Health‐Related Quality of Life.

***p < 0.001, **p < 0.01, *p < 0.05.

**TABLE 3:** Comparisons of psychological variables across demographic and medical characteristics.

		Age (*r*)	Time since diagnosis (*s*)	Time since treatment (*s*)	Diagnosis (*t*)	Stage (*t*)	Scan frequency (*F*)	Race (*t*)
Psychological variables	Scanxiety	−0.141	−0.060	−0.030	0.531	−0.060	0.835	1.616
	FCR severity	0.018	−0.206*	−0.207*	−2.310*	−0.180	0.784	3.448***
	FCR reassurance seeking	0.040	0.194*	0.165	−0.746	−1.006	0.602	−1.908
	Bodily threat monitoring	−0.049	0.013	0.043	−0.551	1.039	0.746	0.483
	Intolerance of uncertainty	−0.226*	0.187	0.285**	−0.342	−0.130	1.546	0.443
	Perceived stress	−0.128	0.121	0.169	0.239	−0.975	2.973	0.998
Health‐related quality of life	HRQoL total	−0.134	0.157	−0.027	2.010*	−1.400	2.350	−1.944
	Physical symptoms	−0.104	0.202*	0.075	2.284*	−0.367	1.341	−1.000
	Psychological symptoms	−0.141	0.086	−0.087	0.585	−1.644	0.823	−1.290
	Existential wellbeing	0.043	0.213*	0.061	1.970	−0.553	1.563	−0.919
	Social support	0.022	0.034	−0.174	1.130	−1.914	0.403	−1.883

*Note:* Statistics reported to 3 decimal places. (*r*) Pearson correlation. (*s*) Spearman's rank correlation. (*t*) *t*‐test (note: negative *t* values for diagnosis represent breast cancer > gynaecological cancer; for stage represent stage2/3 > stage0/1; for race represent other ethnic groups > Caucasian/white). (*F*) One‐way between‐subjects Analysis of Variance (ANOVA). Scan frequency was grouped as: every 1–3 months; every 4–11 months; every year or less.

Abbreviations: FCR, Fear of Cancer Recurrence; HRQoL, Health‐Related Quality of Life.

***p < 0.001, **p < 0.01, *p < 0.05.

### 3.3 Health‐Related Quality of Life (HRQoL) Correlates of Scanxiety and FCR

As seen in Table 2, higher levels of scanxiety were significantly associated with lower overall HRQoL, but at the level of subscales, scanxiety was only significantly associated with existential well‐being and social support subdomains. While greater FCR severity was also significantly associated with lower overall HRQoL, at the level of subscales, FCR severity was only significantly associated with physical and psychological symptom subdomains. Greater scanxiety, but not FCR severity, was also significantly associated with more reassurance‐seeking behaviours.

### 3.4 Shared Mechanisms of Scanxiety and FCR

As seen in Table 2, there was a moderate positive association between scanxiety and FCR severity. As predicted, both FCR and scanxiety were significantly associated with greater intolerance of uncertainty, bodily threat monitoring, and perceived stress at the bivariate level with small‐to‐medium effects across variables, although the effects for FCR severity appear slightly greater.

**FIGURE 2:**
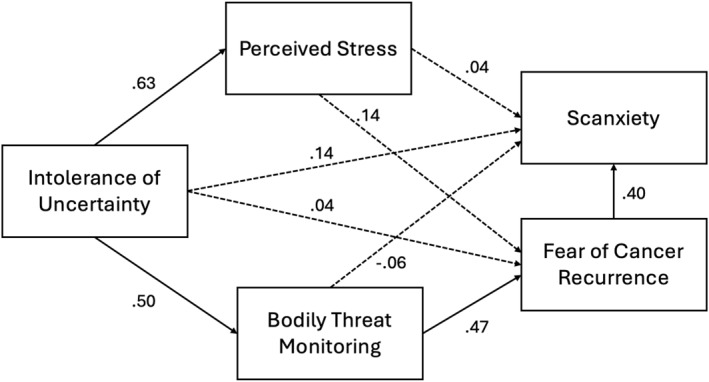
Structural equation model to examine shared mechanisms of FCR and scanxiety. Value for each path represents the standardised estimate (*β*). Solid lines indicate statistically significant paths, *p* ≤ 0.05.

In the structural equation model (Figure 2, Supporting Information S1: Table S2), intolerance of uncertainty was significantly associated with both bodily threat monitoring and perceived stress, but there was no direct relationship with FCR. However, bodily threat monitoring was significantly associated with FCR, and there was a significant indirect effect of intolerance of uncertainty on FCR via bodily threat monitoring [indirect effect: *β* = 0.235, *p* < 0.001] indicating that bodily threat monitoring is a potential intermediate variable in the effect of intolerance of uncertainty on FCR [total effect of intolerance of uncertainty on FCR: *β* = 0.360, *p* < 0.001]. Perceived stress was not significantly associated with FCR and did not function as an intermediate variable in the effect of intolerance of uncertainty on FCR. The a priori model did not include a direct path between FCR and scanxiety (see Supporting Information S1: Table S1 for a priori model indices), however including this theoretically informed pathway improved overall model fit and thus this post‐hoc pathway was reported in the final model results. The initial model did not include a direct pathway between FCR and scanxiety because the focus was on testing shared underlying mechanisms rather than direct effects. However, modelling results indicated a significant direct pathway between FCR and scanxiety, which we incorporated into the final model. This adjustment is consistent with the theoretical possibility that FCR can directly influence scanxiety. In the final model, FCR had a significant direct effect on scanxiety. The final model accounted for 25.4% of variance in bodily threat monitoring, 31.5% of variance in FCR, and 21.4% of variance in scanxiety. An analysis of chi‐square and CFI model fit indices identified that the model had a good fit: *χ*2 = 3.480, *p* = 0.062, CFI = 0.984. Conversely, the TLI (=0.840) and the RMSEA (=0.149, 90%CI: 0.000—0.332, *p* = 0.099) suggested inadequate fit, although this latter finding must be interpreted tentatively as RMSEA is known to over‐reject models in samples smaller than *N* = 200.

## 4 Discussion

In this study, we found that both FCR and scanxiety were prevalent among AYA survivors of breast and gynaecological cancers, even up to 10 years post‐treatment. High proportions of AYA breast and gynaecological cancer survivors reported clinically meaningful (84%) and clinically severe (38%) FCR, underscoring AYAs as a particularly at‐risk group for psychological sequelae post‐cancer treatment [25]. Few previous studies had measured scan‐related anxiety in AYA cancer survivors, thus our findings yield novel data indicating that scanxiety is also common in AYA survivors of breast and gynaecological cancers and may be elevated compared to levels previously reported in other populations [8]. However, the heterogeneity of scanxiety measures used across studies complicates formal comparisons, highlighting the need for standardized scanxiety assessments in future research. Notably, while FCR levels decreased with time since treatment, scanxiety showed no such association, suggesting scanxiety does not simply remit over time. Moreover, AYAs with higher FCR and scanxiety reported poorer health‐related quality of life, emphasizing the need for supportive care interventions targeting these issues to enhance post‐treatment quality of life for younger cancer survivors.

Few previous studies have concurrently assessed FCR and scanxiety, thus the extent to which these constructs overlap in their symptomatology and putative mechanisms has been unclear. We found a moderate correlation between FCR severity and scanxiety in AYA breast and gynaecological cancer survivors. This indicates that while FCR and scanxiety share some overlap, they are also distinct constructs. We also found initial evidence for shared mechanisms of FCR and scanxiety in that both were associated with putative risk and maintenance factors at the bivariate level. Specifically, both were correlated with elevated intolerance of uncertainty, suggesting this could be a dispositional risk factor for both FCR severity and scanxiety, warranting further investigation in longitudinal and experimental studies. Our findings build on a growing evidence base indicating that greater intolerance of uncertainty is associated with heightened FCR in cancer patient and survivor populations [34, 35, 36, 37, 38], including in children [39]. In a study by Savard and colleagues [40], a CBT‐based intervention aimed at reducing FCR also reduced levels of intolerance of uncertainty at 1‐month follow‐up, indicating a possible bi‐directional relationship between FCR and intolerance of uncertainty. There is now robust evidence that individuals who are more intolerant of uncertainty also report greater FCR, and our findings add that they may also report greater scanxiety. Prospective studies examining whether intolerance of uncertainty predicts the maintenance of FCR and scanxiety over time are warranted. There are also novel theories of intolerance of uncertainty in health contexts [15], which could generate novel insights for cancer populations.

We also found that both FCR severity and scanxiety were correlated with greater bodily threat monitoring and perceived stress, suggesting these factors may act as maintenance factors for both. However, only the association between bodily threat monitoring and FCR held in the structural equation model (SEM) which included all correlates together. Additionally, bodily threat monitoring accounted for most of the variance in the link between intolerance of uncertainty and FCR in the SEM, supporting core FCR theory [11, 36]. Interestingly, bodily threat monitoring did not function as an intermediate variable for scanxiety in the SEM. Clinically, this suggests bodily threat monitoring may be less useful in mitigating scanxiety compared to generalised FCR. Surveillance scans provide objective indicators of cancer status, so survivors may be less motivated to closely monitor their bodies during this period than during longer intervals between scans. Bodily threat monitoring is proposed to maintain FCR by increasing the misinterpretation of benign bodily sensations as signs of recurrence or new cancer [16]. Excessive bodily threat monitoring may thus be a potent maintenance factor for prolonged FCR, preventing its resolution over time, especially in patients who seek objective test results to manage their fears. This has implications for clinical care during the post‐treatment surveillance period, as some individuals may resist natural reductions in FCR over time as active surveillance becomes less frequent.

### 4.1 Clinical Implications

In the SEM, perceived stress did not serve as an intermediate variable in the association between intolerance of uncertainty and FCR or scanxiety. Unlike bodily threat monitoring, perceived stress is unlikely a pathway through which intolerance of uncertainty relates to FCR or scanxiety. No significant pathways were observed between putative risk or maintenance factors and scanxiety. Hence, further research is needed to identify mechanisms conferring potential risk for scanxiety. Research into coping mechanisms, metacognitions, and social support is crucial because these factors may influence the way individuals experience and manage scanxiety. Adaptive coping strategies may reduce the emotional impact of regular scans, while maladaptive strategies could heighten distress [18]. Metacognitive beliefs, especially those involving overestimation of risk or lack of control over worry, could amplify anxiety around scan results. Additionally, social support may provide an emotional buffer, helping to mitigate the psychological burden of scan‐related stress. Notably, scans are discussed as fear triggers within some existing FCR interventions [41]. Given the observed association between FCR and scanxiety in this study, these FCR interventions may indirectly reduce scanxiety. However novel, targeted interventions may be needed for scanxiety. Moreover, while FCR interventions encourage survivors to address and accept their fears for long‐term distress reduction, the acute nature of scanxiety may also be ameliorated with distraction strategies; 'flow’ interventions have been identified as promising approaches for mitigating distress during uncertain waiting periods [42].

### 4.2 Study Limitations

The findings of the present study must be qualified by limitations. Principally, this study had a cross‐sectional design which precludes causal interpretation of the effects, including intermediate pathways. Additionally, scanxiety was measured retrospectively, which may have conflated associations with FCR which was measured at the same timepoint. Relatedly, this study focussed on experiences of scanxiety in the run up to scans or medical tests; we know less about the nature and correlates of scanxiety during the acute period of waiting for scan results or the recovery from scanxiety after receiving reassuring news. In addition, over two‐thirds of our sample were breast cancer survivors, highlighting the need for more research on distress mechanisms in young gynaecological cancer survivors. Follow‐up care for gynaecological cancer survivors varies based on the specific type (e.g., ovarian, endometrial, cervical). For example, after treatment for early‐stage endometrial or cervical cancer, follow‐up may involve regular pelvic exams and symptom monitoring rather than annual imaging, unless specific clinical conditions arise. These differences both between and within disease groups likely impact both scanxiety and FCR, and future research assessing the impact of scan frequency and surveillance type would be useful. In addition, this study included AYA post‐treatment survivors; links between FCR and scanxiety and their shared mechanisms may differ for other populations such as older survivors, those receiving active treatment, and those with metastatic disease. Medical information was also self‐reported and could only be verified in participants recruited through clinical pathways. Of note, reassurance‐seeking behaviours were not strongly indicated by theory as a shared mechanism to include in the primary SEM model. However, the observed bivariate associations with scanxiety suggest that this variable may warrant investigation in future research. Notwithstanding these limitations, the present study had several noteworthy strengths. Firstly, this study examined key cancer survivorship issues in an understudied population, that is, AYA cancer survivors. Moreover, this study is one of the first to examine shared mechanisms of scanxiety and FCR, thus shedding light on the relationship between two clinically relevant constructs in cancer survivorship and highlighting the need for further research on novel scanxiety interventions, which may help to reduce the burden of living with and beyond cancer.

## 5 Conclusions

In conclusion, we found that AYA survivors of breast and gynaecological cancers commonly report FCR and scanxiety, which are associated with worse quality of life. While FCR and scanxiety showed some overlap, differences were observed in the psychological processes that may underlie them. Intolerance of uncertainty and bodily threat monitoring are promising mechanisms for reducing FCR and should be measured in future prospective and intervention studies. Further investigation of scanxiety across cancer survivor populations is warranted, using mechanistically informed study designs to identify future therapeutic targets.

## Conflicts of Interest

LCH has previously received consulting fees from Blue Note Therapeutics.

## Supporting information

Supporting Information S1

## Data Availability

The data that support the findings of this study are available from the corresponding author upon reasonable request.
