# Supplementary material for: Scanxiety and Fear of Recurrence in Young Adult Female Breast and Gynaecological Cancer Survivors: Investigating Shared Mechanisms
Source: Psychooncology. 2024 Dec 18;33(12):e70050. doi: 10.1002/pon.70050 (PMC11655430; doi:10.1002/pon.70050)
Supplement: Supplementary file 1 — Supporting Information S1 [file PON-33-e70050-s001.pdf]

Supplemental Tables & Figures

Figure S1. CONSORT Diagram

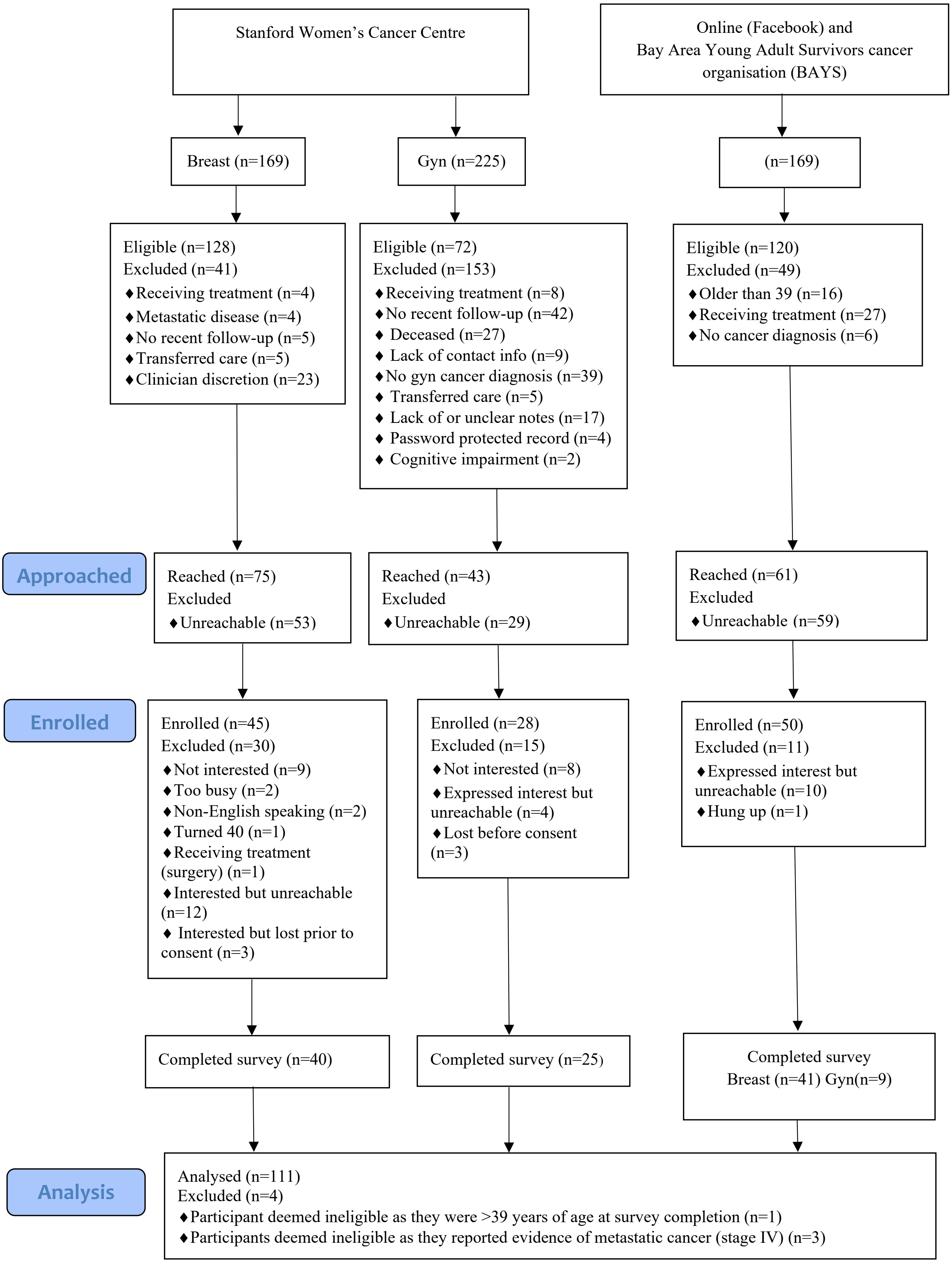

Table S1. Direct effects for the *a priori* structural equation model (without a direct path between FCR and scanxiety).

			Standardised values		
Direct effects	*b*	*SE*	β	95% CI	*p*
IoU → PS	.388	.045	.633	[.510, .731]	< .001
IoU → BTM	.788	.132	.496	[.320, .641]	< .001
IoU → FCR	.024	.072	.038	[-.170, .256]	.713
IoU → Scanxiety	.109	.095	.139	[-.052, .350]	.162
PS → FCR	.143	.107	.138	[-.085, .352]	.222
PS → Scanxiety	.055	.143	.043	[-.208, .292]	.741
BTM → FCR	.190	.037	.474	[.283, .643]	< .001
BTM → Scanxiety	-.032	.054	-.064	[-.310, .207]	.621

*IoU (Intolerance of Uncertainty), FCR (Fear of Cancer Recurrence severity), BTM (Bodily Threat Monitoring), PS (Perceived Stress).

Table S2. Direct effects for the *post-hoc* structural equation model (with a direct path between FCR and scanxiety).

			Standardised values		
Direct effects	*b*	*SE*	β	95% CI	*p*
IoU → PS	.388	.045	.633	[.510, .731]	< .001
IoU → BTM	.788	.132	.496	[.320, .641]	< .001
IoU → FCR	.024	.072	.038	[-.170, .265]	.713
IoU → Scanxiety	.109	.095	.139	[-.052, .350]	.162
PS → FCR	.143	.107	.138	[-.085, .352]	.222
PS → Scanxiety	.055	.143	.043	[-.208, .292]	.741
BTM → FCR	.190	.037	.474	[.283, .643]	< .001
BTM → Scanxiety	-.032	.054	-.064	[-.310, .207]	.621
FCR → Scanxiety	.494	.127	.399	[.140, .588]	.002

*IoU (Intolerance of Uncertainty), FCR (Fear of Cancer Recurrence severity), BTM (Bodily Threat Monitoring), PS (Perceived Stress).
